# Supplementary material for: Disparate impact pandemic framing decreases public concern for health consequences
Source: PLoS One. 2020 Dec 18;15(12):e0243599. doi: 10.1371/journal.pone.0243599 (PMC7748138; doi:10.1371/journal.pone.0243599)
Supplement: S1 Appendix — (PDF) [file pone.0243599.s001.pdf]

S1 Appendix: Experimental texts, images, videos, and other related content

Experimental videos

Experimental videos can be watched on YouTube (equal pandemic, elderly and medical conditions inequality, class inequality). The images and texts used in the videos are presented below.

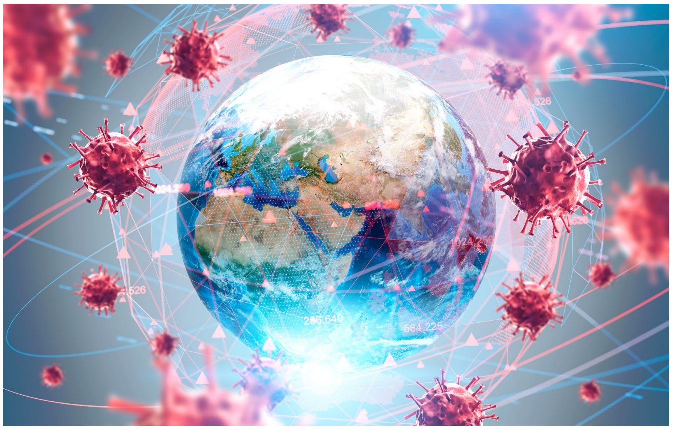

© denisismagilov

We are in the midst of a global disease outbreak. Within a few months after its emergence, the new coronavirus (COVID-19) has spread to almost every country on earth, including the US. Very few events in history have impacted the entirety of humanity in this way, regardless of sex, race, or cultural background.

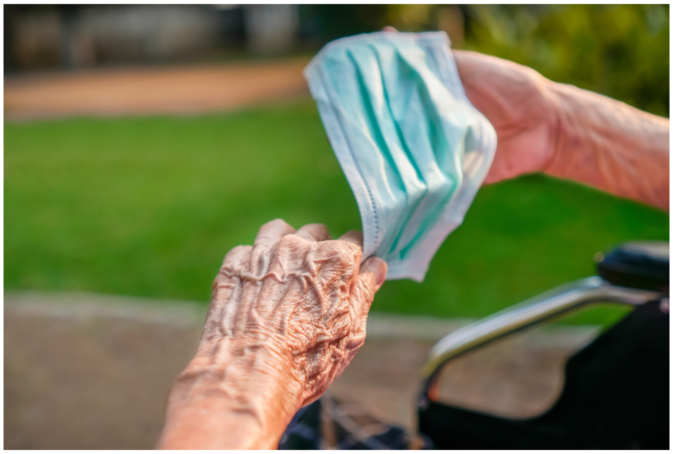

© toa555

The new coronavirus (COVID-19) is not affecting everyone in the same way. The elderly and those with underlying medical conditions such as heart disease, cancer, and diabetes have been disproportionately affected. The number of infections and deaths are significantly higher among this group compared to the rest of the population.

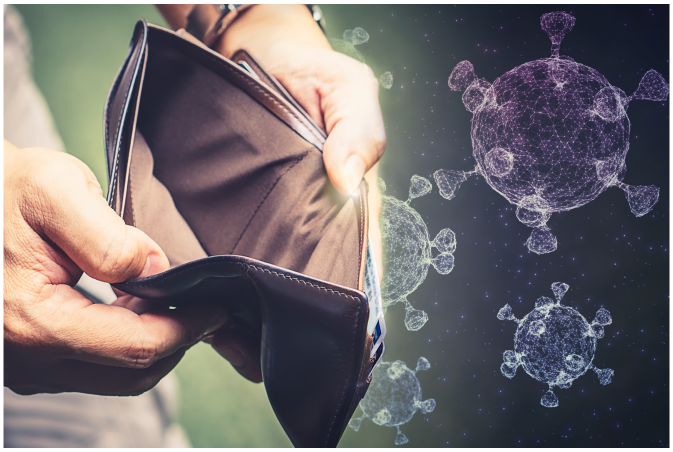

© Chan2545

The new coronavirus (COVID-19) is not affecting everyone in the same way. Poor and low-income communities, particularly minorities such as blacks and Hispanics, have been disproportionately affected. The number of infections and deaths are significantly higher among this group compared to the rest of the population.

Manipulation check

Respondents are asked to answer the following question after watching the video.

Describe in your own words what the text you just listened to was about. [A couple of words or a sentence is enough.]

TEXT ENTRY HERE

Survey questions related to socio-demographic characteristics of respondents

The following socio-demographic questions are asked to respondents prior to answering coronavirus-specific questions. Most of these questions are taken directly from the study by Kuziemko et al [27].

Are you a US resident?

- Yes
- No

In which state do you currently reside?

DROP-DOWN LIST OF STATES HERE

What is your gender?

- Male
- Female
- Other

What is your age? [Enter a number (e.g., 35)]

TEXT ENTRY HERE

What is your marital status?

- Single
- Married

Do you have children living with you?

- Yes
- No

How would you describe your ethnicity/race?

- European American/White

- African American/Black
- Hispanic/Latino
- Asian/Asian American
- Other

How would you describe your religion?

- Christian (Protestant)
- Christian (Catholic)
- Christian (Mormon)
- Christian (Other)
- Jewish
- Muslim
- Hindu
- Buddhist
- Other religion
- No religion

Which category best describes your highest level of education?

- Eighth Grade or Less
- Some High School
- High School Degree/GED
- Some College

- 2-year College Degree
- 4-year College Degree
- Master's Degree
- Doctoral Degree
- Professional Degree (JD, MD, MBA)

What is your current employment status?

- Full-time employee
- Part-time employee
- Self-employed or small business owner
- Unemployed and looking for work
- Student
- Not in labor force (for example: retired, or full-time parent)

What is your occupation?

TEXT ENTRY HERE

What was your TOTAL household income, before taxes, last year?

- $0 - $9,999
- $10,000 - $14,999
- $15,000 - $19,999
- $20,000 - $29,999

- $30,000 - $39,999
- $40,000 - $49,999
- $50,000 - $74,999
- $75,000 - $99,999
- $100,000 - $124,999
- $125,000 - $149,999
- $150,000 - $199,999
- $200,000+

Compared with American families in general today, would you say your family income is above or below average?

- Far below average
- Below average
- Average
- Above average
- Far above average

Which best describes your household's income each month?

- Income is about the same each month
- Income varies somewhat from month to month
- Income varies a lot from month to month

<u>Compared to 10 years ago</u>, do you think your standard of living now is better or worse?

- Much better
- Somewhat better
- About the same
- Somewhat worse
- Much worse

<u>10 years into the future</u>, do you think your standard of living will be better or worse?

- Much better
- Somewhat better
- About the same
- Somewhat worse
- Much worse

On economic policy matters, where do you see yourself on the liberal/conservative spectrum?

- Very conservative
- Conservative
- Moderate
- Liberal
- Very liberal

Generally speaking, do you usually think of yourself as a Republican, a Democrat, an Independent, or what?

- Republican
- Democrat
- Independent
- None

How often do you follow the news?

- Every day
- A few times a week
- Once a week
- Less than once a week
- Never

How much confidence do you have in the <u>scientific community</u>?

- A great deal of confidence
- Only some confidence
- Hardly any confidence at all

Survey questions related to the coronavirus outbreak

After watching the video, respondents were asked to answer the following questions related to coronavirus. Choice ordering was reversed for a random half of respondents in the first five questions.

Do you think the coronavirus is a serious threat to the American people?

- Not a threat at all
- A small threat
- A threat
- A serious threat
- A very serious threat

Do you think it is more important to save lives or to save the economy during this outbreak?

- 1 - Saving lives must be the priority even if it means the economy will suffer
- 2
- 3
- 4
- 5 - Saving the economy must be the priority even if it means lives will be lost

On the whole, how satisfied are you with the way <u>your city</u> has been handling the coronavirus situation?

- Very satisfied

- Fairly satisfied
- Neither satisfied nor dissatisfied
- Not very satisfied
- Not satisfied at all

On the whole, how satisfied are you with the way <u>your state</u> has been handling the coronavirus situation?

- Very satisfied
- Fairly satisfied
- Neither satisfied nor dissatisfied
- Not very satisfied
- Not satisfied at all

On the whole, how satisfied are you with the way <u>the federal government</u> has been handling the coronavirus situation?

- Very satisfied
- Fairly satisfied
- Neither satisfied nor dissatisfied
- Not very satisfied
- Not satisfied at all

How have you been affected by the coronavirus? [Select all that apply.]

- I contracted coronavirus and became ill.
- I lost my job because of coronavirus.
- I experienced a significant decrease in income due to coronavirus.
- I have an underlying medical condition that puts me at greater risk for severe illness.
- Someone in my family contracted coronavirus and became ill.
- Someone in my family lost their job because of coronavirus.
- Someone in my family experienced a significant decrease in income due to coronavirus.
- Someone in my family has an underlying medical condition that puts them at greater risk for severe illness.
- I have not been affected by coronavirus in any major way.
- Other (please specify)

How many days have you been outside in the past seven days?

0

1

2

3

4

5

6

7

Additional variables, conditions

This study is part of a larger project to understand the impact of the coronavirus outbreak on Americans' perceptions of inequality. The survey included many other questions related to respondents' general perceptions regarding opportunity, inequality, and redistribution that are not directly relevant to this paper. The survey also had experimental conditions that are completely unrelated to coronavirus (internet; elderly and medical conditions inequality without reference to coronavirus; class inequality without reference to coronavirus). The researcher is writing another paper in parallel based on these results and is happy to share any materials, data, and/or results if requested.

References

27. Kuziemko, I., M. Norton, E. Saez, and S. Stantcheva. 2015. "How Elastic Are Preferences for Redistribution? Evidence from Randomized Survey Experiments." *American Economic Review* 105(4): 1478-1508.
