# Supplementary material for: Disparate impact pandemic framing decreases public concern for health consequences
Source: PLoS One. 2020 Dec 18;15(12):e0243599. doi: 10.1371/journal.pone.0243599 (PMC7748138; doi:10.1371/journal.pone.0243599)
Supplement: S2 Appendix — (PDF) [file pone.0243599.s002.pdf]

S2 Appendix: Sample size calculations and sample characteristics

Sample size calculations

Sample size calculations were made with the aim of being able to detect a small effect in one-way ANOVA. The `pwr.anova.test` function in the **R** package `pwr` was used for this end. Using this function, and assuming a small effect size (Cohen's *d* = 0.075), a total of 2,250 respondents gives us more than 90% power to be able to detect the effect of interest. (Note that Cohen's *d* is defined as `mu/sigma`, where `mu` is the raw effect size in the original scale and `sigma` is the standard deviation of the outcome variable.) The specific function that was run was this: `pwr.anova.test(k=3, f=0.075, sig.level=0.05, power=0.9).`

Sample characteristics

S2 Table 1 presents sample sizes by condition; since the variable indicating whether the respondent or someone in the respondent's family is at risk is used to show effect heterogeneity in the main text, sample sizes disaggregated by this additional variable are also presented in parentheses. S2 Table 2 presents summary demographics by condition, and S2 Table 3 presents the distribution of respondents across states by condition.

S2 Table 1. Number of respondents by condition.

	Number of respondents

Equal pandemic	870 (not at risk: 597; at risk: 273)
Elderly and medical conditions inequality	880 (not at risk: 641; at risk: 239)
Class inequality	867 (not at risk: 589; at risk: 278)
Total	2,617 (not at risk: 1,827; at risk: 790)

S2 Table 2. Demographics by condition.

	Equal pandemic	Elderly and medical conditions inequality	Class inequality
Age	45.6	44.9	45.8
Gender			
Male	0.452	0.464	0.490
Female	0.539	0.527	0.504
Other	0.009	0.009	0.006
Marital status			
Single	0.509	0.523	0.521
Married	0.491	0.477	0.479
Has children living with them			
No	0.624	0.649	0.645
Yes	0.376	0.351	0.355
Ethnicity/race			
European American/White	0.697	0.667	0.691
African American/Black	0.125	0.119	0.119
Hispanic/Latino	0.091	0.111	0.095
Asian/Asian American	0.053	0.073	0.053
Other	0.035	0.030	0.043
Religion			
Christian (Protestant)	0.268	0.244	0.254
Christian (Catholic)	0.239	0.243	0.255
Christian (Mormon)	0.025	0.017	0.015
Christian (Other)	0.130	0.157	0.137
Jewish	0.032	0.027	0.042
Muslim	0.018	0.014	0.019
Hindu	0.006	0.015	0.003
Buddhist	0.012	0.013	0.008
Other religion	0.048	0.052	0.048
No religion	0.222	0.218	0.219

Highest level of education			
Eighth Grade or Less	0.005	0.003	0.006
Some High School	0.025	0.036	0.017
High School Degree/GED	0.193	0.207	0.203
Some College	0.224	0.232	0.209
2-year College Degree	0.110	0.112	0.104
4-year College Degree	0.264	0.226	0.283
Master's Degree	0.136	0.134	0.131
Doctoral Degree	0.015	0.014	0.020
Professional Degree (JD, MD, MBA)	0.028	0.035	0.028
Employment status			
Full-time employee	0.390	0.417	0.403
Part-time employee	0.106	0.101	0.105
Self-employed or small business owner	0.066	0.066	0.070
Unemployed and looking for work	0.107	0.097	0.105
Student	0.060	0.057	0.058
Not in labor force (for example: retired, or full-time parent)	0.272	0.262	0.260
Total household income before taxes			
$0 - $9,999	0.069	0.076	0.060
$10,000 - $14,999	0.064	0.049	0.039
$15,000 - $19,999	0.055	0.047	0.053
$20,000 - $29,999	0.087	0.103	0.103
$30,000 - $39,999	0.107	0.094	0.125
$40,000 - $49,999	0.093	0.089	0.093
$50,000 - $74,999	0.184	0.195	0.183
$75,000 - $99,999	0.139	0.122	0.128
$100,000 - $124,999	0.064	0.077	0.075
$125,000 - $149,999	0.055	0.061	0.047
$150,000 - $199,999	0.047	0.057	0.044
$200,000+	0.035	0.030	0.050
Income volatility			
Income is about the same each month	0.634	0.611	0.612
Income varies somewhat from month to month	0.282	0.280	0.293
Income varies a lot from month to month	0.084	0.109	0.095
Liberal/conservative spectrum			
Very conservative	0.113	0.130	0.104
Conservative	0.205	0.188	0.204
Moderate	0.410	0.432	0.443
Liberal	0.186	0.176	0.153
Very liberal	0.086	0.075	0.096
Party identity			
Republican	0.330	0.318	0.343
Democrat	0.393	0.375	0.322
Independent	0.236	0.234	0.263
None	0.041	0.073	0.073

Frequency of following news			
Never	0.025	0.030	0.022
Less than once a week	0.084	0.073	0.077
Once a week	0.095	0.103	0.116
A few times a week	0.240	0.275	0.268
Every day	0.555	0.519	0.517
Confidence in the scientific community			
Hardly any confidence at all	0.070	0.080	0.087
Only some confidence	0.437	0.436	0.449
A great deal of confidence	0.493	0.484	0.465

Age is in years. All other numbers presented are proportions.

S2 Table 3. Number of respondents in each state by condition.

State	Equal pandemic	Elderly and medical conditions inequality	Class inequality
Alabama	12	14	18
Alaska	1	1	2
Arizona	14	26	20
Arkansas	10	8	6
California	95	87	86
Colorado	14	15	12
Connecticut	6	14	12
Delaware	4	4	1
District of Columbia	4	3	3
Florida	71	76	85
Georgia	21	29	33
Hawaii	3	4	2
Idaho	7	1	4
Illinois	41	41	37
Indiana	32	10	7
Iowa	5	8	6
Kansas	8	3	10
Kentucky	12	13	6
Louisiana	13	7	10
Maine	5	5	5
Maryland	9	22	19
Massachusetts	22	16	14
Michigan	22	35	22
Minnesota	14	8	14
Mississippi	10	8	3
Missouri	11	16	9
Montana	3	1	5
Nebraska	5	4	2

Nevada	7	10	13
New Hampshire	2	3	2
New Jersey	28	33	31
New Mexico	5	6	6
New York	76	54	77
North Carolina	26	23	28
North Dakota	0	2	0
Ohio	23	23	27
Oklahoma	6	4	13
Oregon	15	13	9
Pennsylvania	49	51	36
Rhode Island	4	4	1
South Carolina	19	17	10
South Dakota	2	1	0
Tennessee	13	16	15
Texas	47	75	67
Utah	5	8	4
Vermont	1	1	2
Virginia	23	24	31
Washington	17	18	24
West Virginia	7	0	4
Wisconsin	19	12	14
Wyoming	2	1	0
Respondent does not reside in the United States	0	2	0
Total	870	880	867
