# Supplementary material for: Disparate impact pandemic framing decreases public concern for health consequences
Source: PLoS One. 2020 Dec 18;15(12):e0243599. doi: 10.1371/journal.pone.0243599 (PMC7748138; doi:10.1371/journal.pone.0243599)
Supplement: S3 Appendix — (PDF) [file pone.0243599.s003.pdf]

S3 Appendix: Regression results

S3 Table 1 presents linear regression results for the outcome "coronavirus serious threat," while S3 Table 2 presents linear regression results for the outcome "economy must be saved." S3 Table 3 presents additional results for these outcomes based on ordinal logistic regression models. S3 Table 4 presents estimates disaggregated by respondent's at risk status, and S3 Table 5 presents estimates based on models that include interactions between the at-risk variable and the experimental conditions. Finally, S3 Table 6 presents estimates from models that treat the two inequality conditions as the reference category to report an effect estimate for the equal pandemic condition, and S3 Table 7 presents results from additional outcomes related to respondent's level of satisfaction with the way their city, state, and the federal government has been handling the coronavirus situation.

S3 Table 1. Coronavirus serious threat.

Models w/o any demographic covariates

	Coefficient estimate	Standard error	p-value
Elderly and medical conditions inequality	-0.166	0.052	0.001
Class inequality	-0.067	0.052	0.199

Models w/ demographic covariates

	Coefficient estimate	Standard error	p-value
Elderly and medical conditions inequality	-0.141	0.046	0.002
Class inequality	-0.019	0.046	0.689

S3 Table 2. Economy must be saved.

Models w/o any demographic covariates

	Coefficient estimate	Standard error	p-value

Elderly and medical conditions inequality	0.201	0.062	0.001
Class inequality	0.138	0.062	0.027

Models w/ demographic covariates

	Coefficient estimate	Standard error	p-value
Elderly and medical conditions inequality	0.182	0.058	0.002
Class inequality	0.092	0.058	0.112

S3 Table 3. Estimates from the ordinal logistic regression models.

	Coronavirus serious threat	Economy must be saved
Elderly and medical conditions inequality	-0.261 (0.088)**	0.286 (0.086)***
Class inequality	-0.130 (0.088)	0.194 (0.087)*

Coefficient estimates are in log-odds. The numbers inside the parentheses are standard errors. Estimates are based on models without any demographic covariates. Stars denote p-values: ˙ p<0.1, * p<0.05, ** p<0.01, *** p<0.001.

S3 Table 4. Estimates disaggregated by respondent's at risk status.

At risk

	Coronavirus serious threat	Economy must be saved
Elderly and medical conditions inequality	-0.061 (0.082)	0.101 (0.108)
Class inequality	-0.071 (0.079)	0.089 (0.104)

Not at risk

	Coronavirus serious threat	Economy must be saved
Elderly and medical conditions inequality	-0.184 (0.064)**	0.226 (0.075)**
Class inequality	-0.068 (0.065)	0.163 (0.077)*

The numbers inside the parentheses are standard errors. Estimates are based on models without any demographic covariates. Stars denote p-values: ˙ p<0.1, * p<0.05, ** p<0.01, *** p<0.001.

S3 Table 5. Estimates from models with interactions.

	Coronavirus serious threat	Economy must be saved
Elderly and medical conditions inequality	-0.184 (0.061)**	0.226 (0.073)**

Class inequality	-0.068 (0.062)	0.163 (0.075)*
At risk	0.358 (0.078)***	-0.214 (0.094)*
Elderly and medical conditions inequality x At risk	0.123 (0.112)	-0.124 (0.136)
Class inequality x At risk	-0.002 (0.110)	-0.075 (0.133)

The numbers inside the parentheses are standard errors. Estimates are based on models without any demographic covariates. Stars denote p-values: ˙ p<0.1, * p<0.05, ** p<0.01, *** p<0.001.

S3 Table 6. Equal pandemic effect.

	Coronavirus serious threat	Economy must be saved
Equal pandemic	0.117 (0.045)**	-0.170 (0.054)**

The numbers inside the parentheses are standard errors. Estimates are based on models without any demographic covariates. Stars denote p-values: ˙ p<0.1, * p<0.05, ** p<0.01, *** p<0.001.

S3 Table 7. Other outcomes.

	Satisfied with city	Satisfied with state	Satisfied with federal govt
Elderly and medical conditions inequality	0.028 (0.052)	-0.039 (0.059)	0.035 (0.065)
Class inequality	-0.044 (0.053)	-0.111 (0.059)˙	-0.060 (0.065)

The numbers inside the parentheses are standard errors. Estimates are based on models without any demographic covariates. Stars denote p-values: ˙ p<0.1, * p<0.05, ** p<0.01, *** p<0.001.
