# Supplementary material for: Disparate impact pandemic framing decreases public concern for health consequences
Source: PLoS One. 2020 Dec 18;15(12):e0243599. doi: 10.1371/journal.pone.0243599 (PMC7748138; doi:10.1371/journal.pone.0243599)
Supplement: Attachment — Submitted filename: PLOS-ONE-Response-to-Reviewers.docx [file pone.0243599.s006.docx]

September 22, 2020

We thank the editor and the reviewers for their constructive and helpful comments. We carefully reviewed the points raised by all four reviewers and revised the manuscript accordingly. Below, you can find our responses organized into four sections -- one for each reviewer.

**Reviewer #1**

“For instance, when I was reading I thought it would be good to remind the reader of what ‘natural inequality’ means when discussing the findings (term mentioned on page 4 but not again until the end of page 7).”

*Author’s response:* We added a sentence in parentheses right after we mention ‘natural inequality’ for the first time in the main findings subsection (p. 9), reminding the reader what ‘natural inequality’ means in this context.

“Page 8 ‘main findings’—first paragraph is how the variables were labeled/defined, why is this in the findings section and not in the methods section?”

*Author’s response:* We removed this paragraph from the results section and moved the information provided to the methods subsection on experimental design (p. 5).

“Also on page 8—I would mention what ‘manipulation check’ is for readers who may not know exactly what this means.”

*Author’s response:* We added a sentence defining what ‘manipulation check’ means (p. 8).

“Page 9, second paragraph, I think it is informal when using ‘the first outcome’—I suggest that the outcome be defined and explicitly stated and interpreted to make sure the reader does not have to scroll back up to another section of the paper to know what that outcome is and what it means—this makes the paragraphs in the main findings section hard to follow--I would suggest being much more explicit throughout.”

*Author’s response:* We revised the wording accordingly to avoid the first/second outcome language (pp. 8-11).

“Where is the rational for why subjects were randomized the way that they were (end of page 4)? Why would elderly and those with medical conditions be one condition? We know that low income and poor populations are more likely to have medical conditions (diabetes, cardiovascular disease, obesity, COPD) that arises from systemic oppression and racism, so why separate poor and low income from medical conditions when they are related as much as being elderly can be related with medical conditions? I would definitely add text on how randomization occurred—decision process and theory of why/how these groups were created. Also, if there is a way to perform some sensitivity analyses which could show us what the results would look like if one group was just elderly, and the other group was low-income and with medical conditions, I would highly suggest including this—at the very least, provide some discussion on this in the manuscript.”

*Author’s response:* Our justification for having separate conditions for natural inequality and class inequality is that this study is primarily concerned with how people understand the impact of the pandemic, not about the actual facts. While it is certainly true that the poor and minorities are more likely to have medical conditions as a matter of science, we tried to formulate treatment conditions that reflect the ways that the pandemic is discussed in public discourse. We added a sentence in the experimental design subsection that mentions this justification (p. 4). Furthermore, we are discussing this very important point raised by the reviewer under the newly added limitations section at the end of the manuscript (pp. 12-13).

**Reviewer #2**

“In results you provide a robust description of the data on the use of the class inequality and also the use of the natural inequality. In the discussion there is less or no explanation of the data in results on the class inequality. Please discuss the class inequality data in discussion in some way.”

*Author’s response:* We added a paragraph on ‘class inequality’ in the discussion section (p. 12). We discuss a possible explanation of the null class effect in relation to the highly politicized nature of issues related to class and race in the US, which makes it difficult to move people’s opinions on these issues compared to more neutral and directly health-related topics such as the elderly and those with medical conditions.

“Please explain how the custom script was developed and whether it was validated in some way by having another person read it or whether it was pre-tested on a sample before its use. There is not section on limitations. The development of the custom script might be a limitation.”

*Author’s response:* The custom script that is referred to in the manipulation checks subsection was developed by the researchers alone after the sample was gathered (it is available as part of the replication files). What the script does is that it takes in the survey answer given by the respondent to the manipulation check question --- “Describe in your own words what the text you just listened to was about. [A couple of words or a sentence is enough.” --- and checks whether the answer mentions words relevant to the video watched. For example, if the respondent watched the ‘natural inequality’ condition, we expect the respondent to mention words such as “elderly,” “vulnerable”, “medical condition,” or some other indication that the respondent actually watched the video. We completely agree that the ad-hoc nature of this script is a limitation, and we are adding this point to the newly added limitations section (pp. 12-13).

If, by custom script, the reviewer is instead referring to the development of the experimental texts that are narrated to respondents as part of the survey, these texts were written by the researchers themselves after a careful reading of relevant news articles and scientific communications (p. 6). In addition, the researchers received feedback from colleagues when deciding on the particular wording to be used in these texts.

“Also can you go to greater lengths in the discussion to clarify second and first outcomes as described in this section below- just make it clearer rather then simply saying the first outcome or the second outcome. I had to keep checking back to remind myself of these outcomes. ‘While the natural inequality condition led to significant changes in both outcomes, class inequality condition was weaker in its effects. Despite the effect being in the same direction as natural inequality, class inequality led to significant changes only in the second outcome’ (second outcome was what again?) so were people who had the class inequity frame more or less likely to feel threatened by the virus and how did they feel about saving the economy?”

*Author’s response:* We revised the wording accordingly to avoid the first/second outcome language (this point was raised by another reviewer as well) (pp. 8-11). It should now be much easier to follow which effect is discussed as well as the directionality of the effect.

**Reviewer #3**

“Methods---The authors noted the use of linear regression modeling. However, their outcome variables are measured on a Likert Scale and as such are strictly ordinal variables. Did they test for violations of OLS assumptions? Perhaps they can consider ordinal logistic regression (especially if the assumptions of OLS are violated).”

*Author’s response:* We ran additional analyses (see r&r-additional-analyses.r which is available as part of the replication files) to test for violations of the OLS assumptions. Not surprisingly, we found evidence for the violation of the normality of errors assumption. While this assumption is not necessary for OLS regression to be BLUE (best linear unbiased estimator), it does impact standard errors and p-values. As a result, we fit additional ordinal logistic regression models, which did not change our substantive results: the ‘natural inequality’ condition decreases the log-odds of selecting a higher threat category by -0.261 (p-value=0.003), while the ‘class inequality’ condition decreases the log-odds of selecting a higher threat category by -0.130 (p-value=0.139). Similarly, the ‘natural inequality’ condition increases the log-odds of selecting a category in the direction of ‘the economy must be saved’ by 0.286 (p-value<0.001), while the ‘class inequality’ condition increases the log-odds of selecting a category in the direction of ‘the economy must be saved’ by 0.194 (p-value=0.026). Given that our substantive results remain unchanged regardless of whether we use OLS regression or ordinal logistic regression, we decided to keep the OLS results in the main manuscript (because they are much simpler to comprehend and communicate) while at the same time including the ordinal logistic regression results in S3 Appendix as an additional robustness check. We revised the main text to refer the reader to S3 Appendix for these additional results (p. 8).

“Discussion---One of the more interesting findings of this study is that a focus on the disproportionate negative impact of the virus on the elderly (and NOT on poor and low-income communities) minimizes people’s perception of the threat of the virus. Why is this so? While the authors discuss the ‘elderly finding’, the finding (or the lack of finding) for the poor and low-income arm is not discussed at all. This is an important omission that needs to be addressed.”

*Author’s response:* We added a paragraph on ‘class inequality’ in the discussion section (this point was raised by another reviewer as well) (p. 12). We discuss a possible explanation of the null class effect in relation to the highly politicized nature of issues related to class and race in the US, which makes it difficult to move people’s opinions on these issues compared to more neutral and directly health-related topics such as the elderly and those with medical conditions.

**Reviewer #4**

“The author should be much clearer about whether the treatment group is manipulating class inequality or race in the inequality frame. I pulled the supplementary materials to find the text of this manipulation and it is definitely a conflation of class and race (‘poor and low-income communities, particularly minorities such as blacks and Hispanics, have been disproportionately affected’). The paper starts by discussing this as just class inequality, but occasionally also discusses race. There is a very broad literature in social science on group-cues (see e.g., [Nelson and Kinder 1996](https://www.journals.uchicago.edu/doi/abs/10.2307/2960149)) that is not discussed at all. This is curious, as sociology, political science, and communication all have spent much attention on looking at how framing information in terms of the groups affected – particularly as it relates to class and race – matters. More attention to framing and race/class would be warranted in this paper.”

*Author’s response:* The ‘class inequality’ condition indeed refers to both class and race factors, and we revised the manuscript to make this clearer; the condition is now called ‘class and race inequality’ (p. 4 and elsewhere). We also further clarified in the experimental design subsection that the conditions are chosen to reflect the ways that the pandemic is discussed in public discourse, and, in particular, class and race disadvantage is included in the same treatment because this is usually the way the discussion is framed in public discourse (p. 4). Finally, we are discussing this highly important point under limitations since the study does not separately look at class and race (pp. 12-13).

“Neither the background nor the methods section motivates or pre-specifies the treatment effect heterogeneity findings – among those not at risk and with no family at risk. Since the authors present these results, they should explain why they sought to look at the data by these strata earlier in the manuscript.”

*Author’s response:* We added a sentence in the experimental design subsection that foreshadows why the “at risk” variable is particularly valuable and relevant in this context (pp. 5-6).

“Did the authors pre-register their hypotheses and study design?”

*Author’s response:* We pre-registered the design -- including all of the variables discussed in the text -- but not the specific hypotheses tested here. While hypotheses not pre-registered could potentially suffer from the multiple comparisons problem, the p-values associated with the ‘natural inequality’ condition is sufficiently small (p<0.001 or close to that level) and would continue to be significant at the alpha=0.05 level even with adjustments made for multiple testing (e.g., FDR or Bonferroni correction). We are also adding a sentence in the experimental design subsection noting that the design is pre-registered but not the hypotheses (p. 6).

“You report separate models stratified by risk as noted above, but it would also be statistically sound to estimate an interaction term by at-risk vs. not-at risk and the treatment groups. The way the results by subgroup are reported on p. 9 is confusing -- it sounds like the authors are reporting the coefficients on the risk variable and not the treatment groups? I recommend revising the discussion of the treatment effect heterogeneity section so it is more clear and also reports the results of the models estimated with interaction terms.”

*Author’s response:* We revised the manuscript to clarify the procedure followed to demonstrate effect heterogeneity (pp. 9-10). This procedure involves, for a given outcome, fitting two separate regressions of the outcome on the experimental conditions, one on the at-risk sub-sample and the other on the not-at-risk sub-sample. Then, from these two models, we can get two separate estimates of the ‘natural inequality’ effect based on the two sub-samples. The point made in the manuscript is that the ‘natural inequality’ effect estimate based on the not-at-risk sub-sample is more than twice as large (sometimes close to 3 times) as that of the estimate based on the at-risk sub-sample, which indicates that the ‘natural inequality’ effect is especially strong among this not-at-risk group (see S3 Table 4 below).

In addition to this procedure, upon the reviewer’s request, we fitted pooled models with interactions between the experimental conditions and the at-risk variable (outcome ~ at_risk + natural_ineq + class_ineq + at_risk * natural_ineq + at_risk * class_ineq). The coefficient estimates on the interactions from these models are insignificant (see S3 Table 5 below), which is not surprising because the experiment is not powered to be able to detect interaction effects. Given that (i) interactions inherently require larger sample sizes to be able to achieve a certain level of statistical power and (ii) the interaction effect in this case is smaller (about half the size) of the main effect, having enough statistical power to be able to detect a significant interaction effect in this case would require a sample size approximately 16 times as large ([Gelman 2018](https://statmodeling.stat.columbia.edu/2018/03/15/need-16-times-sample-size-estimate-interaction-estimate-main-effect/)). While the interaction effect is not significant (yet it’s in the expected direction), we believe that there is still value in discussing the drastic difference between the magnitude of the treatment effects in the two sub-samples (at-risk vs not-at-risk), especially because (i) the difference can be justified in relation to theory as covered in the discussion section and (ii) the at-risk main effect is significant and both the at-risk main effect and interactions are in the expected direction (i.e., opposite of the treatment effects), which explains why the treatment effects are drastically smaller in the at-risk sub-sample. We are adding a discussion of the models with interactions in the main findings subsection as well as referring the reader to the newly added results presented in S3 Appendix (p. 10).

All of these being said, if the editor or the reviewer has a strong preference against the inclusion of these effect heterogeneity results in the manuscript in light of these additional results, we would be more than happy to remove the effect heterogeneity discussion entirely from the paper or move it to an appendix.

**S3 Table 4. Estimates disaggregated by respondent’s at risk status.**

*At risk*

|  | Coronavirus serious threat | Economy must be saved |
| --- | --- | --- |
| Natural inequality | -0.061 (0.082) | 0.101 (0.108) |
| Class and race inequality | -0.071 (0.079) | 0.089 (0.104) |

*Not at risk*

|  | Coronavirus serious threat | Economy must be saved |
| --- | --- | --- |
| Natural inequality | -0.184 (0.064)** | 0.226 (0.075)** |
| Class and race inequality | -0.068 (0.065) | 0.163 (0.077)* |

The numbers inside the parentheses are standard errors. Estimates are based on models without any demographic covariates. Stars denote p-values: ˙ p<0.1, * p<0.05, ** p<0.01, *** p<0.001.

**S3 Table 5. Estimates from models with interactions.**

|  | Coronavirus serious threat | Economy must be saved |
| --- | --- | --- |
| Natural inequality | -0.184 (0.061)** | 0.226 (0.073)** |
| Class and race inequality | -0.068 (0.062) | 0.163 (0.075)* |
| At risk | 0.358 (0.078)*** | -0.214 (0.094)* |
| Natural inequality x At risk | 0.123 (0.112) | -0.124 (0.136) |
| Class and race inequality x At risk | -0.002 (0.110) | -0.075 (0.133) |

The numbers inside the parentheses are standard errors. Estimates are based on models without any demographic covariates. Stars denote p-values: ˙ p<0.1, * p<0.05, ** p<0.01, *** p<0.001.

“Authors describe the ‘equal pandemic’ frame as the control, but I would argue that most news coverage emphasizes groups at risk rather than the idea of ‘equal pandemic’. By using the ‘equal pandemic’ as the omitted/reference category they assume in their interpretation that the inequality treatments are what are driving the results – but given there is no ‘no media exposure’ control group, it could be that the ‘equal pandemic’ is actually the distinct frame. Authors might want to comment on this in the discussion. Looking at the figures, for instance, I would actually not use the term ‘control’ but instead call this condition ‘Equal pandemic’.”

*Author’s response:* We revised the text/figures to refer to the “equal pandemic” condition as “equal pandemic” rather than control (p. 4 and elsewhere). We also added a note in the “Overview of statistical models used” sub-section that the choice of reference category in this case is somewhat arbitrary (p. 7). Finally, we added a new table of results in S3 Appendix based on models that treat the two inequality conditions as the reference category to estimate an equal pandemic effect.
