# Supplementary material for: Disparate impact pandemic framing decreases public concern for health consequences
Source: PLoS One. 2020 Dec 18;15(12):e0243599. doi: 10.1371/journal.pone.0243599 (PMC7748138; doi:10.1371/journal.pone.0243599)
Supplement: Attachment — Submitted filename: PLOS-ONE-Response-to-Reviewers-Round-2.docx [file pone.0243599.s007.docx]

November 21, 2020

I thank the editor and the reviewers for their constructive and helpful comments. As suggested by the editor, I carefully reviewed the points raised in the Editor Comments section and revised the manuscript accordingly. Please see my responses below.

“Please change ‘natural inequality’ to a more descriptive term representing that construct such as ‘elderly and medical conditions inequality’ in the text and tables/figures.”

*Author’s response:* I changed ‘natural inequality’ to ‘elderly and medical conditions inequality’ everywhere in the text, tables, and figures.

“There is still confusion about whether racial/ethnic minorities are a focal group or not. It’s clear the data exist to analyze this population from the survey instrument but the analysis seems to combine this population with low income persons. If racial/ethnic minorities are not a focus and not genuinely analyzed, then it might be better to drop this reference in the text and tables/figures and instead list this as a limitation and an area of future work.”

*Author’s response:* I removed all references to race and ethnicity in the main text, tables, and figures. In particular, I changed the ‘class and race inequality’ condition to ‘class inequality’ and removed any discussion of minorities in the main text. I also revised the Limitations section to discuss how the ‘class inequality’ condition combines class and race axes into one treatment and that investigating how these two axes intersect would be a fruitful area of future work.

“The paper would benefit from revising the language in the first paragraph of the Experimental Design, especially clearly defining the three groups. If the ‘equal pandemic’ is indeed the control in the experiment, then a more detailed explanation of that group is needed.”

*Author’s response:* I revised the first paragraph of the Experimental Design section to describe the three groups in more detail. In particular, I defined the “equal pandemic” framing in the following way:

(1) the “equal pandemic” framing, which does not say anything about the disparate impact of the pandemic on different groups but instead emphasizes how the outbreak has been affecting everyone regardless of their background

“Please add a table 2 x 2 perhaps with the treatment on one side of the table and outcomes across the top as suggested by reviewer 2.”

*Author’s response:* I added a table to the main text (Table 1) that presents the estimates for “elderly and medical conditions inequality” and “class inequality” (along the rows) for the two outcomes (along the columns). I refer the reader to this table in the Main Findings subsection.
